# Toxic Shock Syndrome Toxin 1 Evaluation and Antibiotic Impact in a Transgenic Model of Staphylococcal Soft Tissue Infection

**DOI:** 10.1128/mSphere.00665-19

**Published:** 2019-10-09

**Authors:** Hema Sharma, Claire E. Turner, Matthew K. Siggins, Mona El-Bahrawy, Bruno Pichon, Angela Kearns, Shiranee Sriskandan

**Affiliations:** a Department of Infectious Disease, Imperial College London, London, United Kingdom; b Department of Histopathology, Imperial College London, United Kingdom; c National Infection Service, Public Health England, London, United Kingdom; University of Nebraska Medical Center

**Keywords:** antibiotics, dissemination, HLA-DQ8, nonmenstrual toxic shock syndrome, *Staphylococcus aureus*, TSST-1, transgenic mice

## Abstract

Nonmenstrual toxic shock syndrome (nmTSS), linked to TSST-1-producing CC30 Staphylococcus aureus, is the leading manifestation of toxic shock syndrome (TSS). Due to case rarity and a lack of tractable animal models, TSS pathogenesis is poorly understood. We developed an S. aureus abscess model in HLA class II transgenic mice to investigate pathogenesis and treatment. TSST-1 sensitivity was established using murine spleen cell proliferation assays and cytokine assays following TSST-1 injection *in vivo*. HLA-DQ8 mice were infected subcutaneously with a *tst*-positive CC30 methicillin-sensitive S. aureus clinical TSS-associated isolate. Mice received intraperitoneal flucloxacillin, clindamycin, flucloxacillin and clindamycin, or a control reagent. Abscess size, bacterial counts, TSST-1 expression, and TSST-1 bioactivity were measured in tissues. Antibiotic effects were compared with the effects of control reagent. Purified TSST-1 expanded HLA-DQ8 T-cell Vβ subsets 3 and 13 *in vitro* and instigated cytokine release *in vivo*, confirming TSST-1 sensitivity. TSST-1 was detected in abscesses (0 to 8.0 μg/ml) and draining lymph nodes (0 to 0.2 μg/ml) of infected mice. Interleukin 6 (IL-6), gamma interferon (IFN-γ), KC (CXCL1), and MCP-1 were consistent markers of inflammation during infection. Clindamycin-containing antibiotic regimens reduced abscess size and TSST-1 production. Infection led to detectable TSST-1 in soft tissues, and TSST-1 was detected in draining lymph nodes, events which may be pivotal to TSS pathogenesis. The reduction in TSST-1 production and lesion size after a single dose of clindamycin underscores a potential role for adjunctive clindamycin at the start of treatment of patients suspected of having TSS to alter disease progression.

**IMPORTANCE** Staphylococcal toxic shock syndrome (TSS) is a life-threatening illness causing fever, rash, and shock, attributed to toxins produced by the bacterium Staphylococcus aureus, mainly toxic shock syndrome toxin 1 (TSST-1). TSS was in the past commonly linked with menstruation and high-absorbency tampons; now, TSS is more frequently triggered by other staphylococcal infections, particularly of skin and soft tissue. Investigating the progress and treatment of TSS in patients is challenging, as TSS is rare; animal models do not mimic TSS adequately, as toxins interact best with human immune cells. We developed a new model of staphylococcal soft tissue infection in mice producing human immune cell proteins, rendering them TSST-1 sensitive, to investigate TSS. The significance of our research was that TSST-1 was found in soft tissues and immune organs of mice and that early treatment of mice with the antibiotic clindamycin altered TSST-1 production. Therefore, the early treatment of patients suspected of having TSS with clindamycin may influence their response to treatment.

## Abstract (precis)

Staphylococcal toxic shock syndrome (TSS) is a life-threatening illness causing fever, rash, and shock, attributed to toxins produced by the bacterium Staphylococcus aureus, mainly toxic shock syndrome toxin 1 (TSST-1). TSS was in the past commonly linked with menstruation and high-absorbency tampons; now, TSS is more frequently triggered by other staphylococcal infections, particularly of skin and soft tissue. Investigating the progress and treatment of TSS in patients is challenging, as TSS is rare; animal models do not mimic TSS adequately, as toxins interact best with human immune cells. We developed a new model of staphylococcal soft tissue infection in mice producing human immune cell proteins, rendering them TSST-1 sensitive, to investigate TSS. The significance of our research was that TSST-1 was found in soft tissues and immune organs of mice and that early treatment of mice with the antibiotic clindamycin altered TSST-1 production. Therefore, the early treatment of patients suspected of having TSS with clindamycin may influence their response to treatment.

## INTRODUCTION

Staphylococcal toxic shock syndrome (TSS) is a potentially lethal illness characterized by fever, rash, desquamation, organ dysfunction, and shock. The syndrome is attributed to superantigens produced by Staphylococcus aureus, in particular, toxic shock syndrome toxin 1 (TSST-1). TSST-1 has been associated with almost all menstrual TSS (mTSS) and half of nonmenstrual TSS (nmTSS) cases (1), while staphylococcal enterotoxins A, B, and C (SEA, SEB, and SEC) are implicated in the remaining nmTSS cases (2, 3). Superantigens bind simultaneously to the HLA class II molecule on antigen-presenting cells and the T-cell receptor, causing massive T-cell activation, expansion, and cytokine release (2). In the United Kingdom, nmTSS is now more common than mTSS. Skin and soft tissue infections (SSTI) are the most frequent trigger for nmTSS, which, in the United Kingdom, is associated with TSST-1-producing strains in 41% of cases (4). Due to its rarity, TSS pathogenesis is poorly understood, and there is a paucity of clinical data to guide treatment choices. Notwithstanding a lack of clinical trial or *in vivo* data, combination antimicrobial treatment with β-lactams and protein synthesis inhibitors is recommended for staphylococcal TSS (5), based solely upon *in vitro* studies and extrapolation from observational studies of streptococcal TSS.

Murine models of TSS may provide insight into TSS pathogenesis and antimicrobial efficacy but are hampered by low-affinity interactions between murine major histocompatibility class II (MHC II) and staphylococcal superantigens. Prior sensitization with lipopolysaccharide (6) or d-galactosamine (7) has been used to induce superantigen-mediated lethality, though the pathological changes incurred may differ markedly from those induced by superantigen alone. Transgenic expression of human HLA class II can render mice superantigen sensitive and allows investigation of superantigen-associated inflammation without the need for sensitization (8), removing potential experimental confounders. There are few recent studies of staphylococcal TSS infection using contemporary clinical strains and none that evaluate disease progression and toxin release in superantigen-sensitive mice. We developed a humanized transgenic model of superantigen-associated SSTI using a clinical TSST-1-producing CC30 methicillin-sensitive S. aureus (MSSA) TSS-associated isolate to investigate the pathogenesis and treatment of nmTSS.

(This work was presented in part at the 55th Interscience Conference on Antimicrobial Agents and Chemotherapy [ICAAC], San Diego, CA, in September 2015.)

## RESULTS

### HLA-DQ8 transgenic mice are superantigen and TSST-1 sensitive.

**FIG 1:**
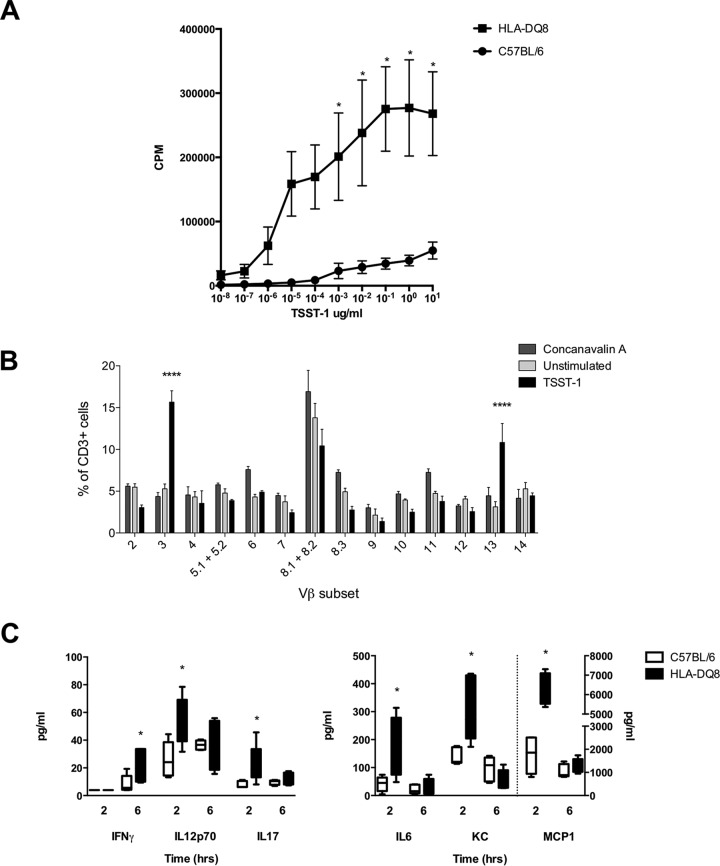
HLA-DQ8 mice are superantigen sensitive. (A) Sensitivities of HLA-DQ8 and C57BL/6 splenocytes to TSST-1. Splenocytes (1 × 10^6^/ml) from HLA-DQ8 and C57BL/6 mice were exposed to 0 to 10 μg/ml of TSST-1. Proliferation was measured by [^3^H]thymidine uptake. CPM, counts per minute. Proliferation in the presence of 5 μg/ml concanavalin (positive control) was 229,806 ± 48,570 CPM for C57BL/6 spleen cells and 373,349 ± 56,008 CPM for HLA-DQ8 spleen cells. Data are means ± SD of results from three individual mice. *, *P* < 0.05; **, *P* < 0.01 between HLA-DQ8 and C57BL/6 by ANOVA. (B) Percentages of spleen cells from HLA-DQ8 mice in each CD3ε^+^ TCR Vβ subset expanded by TSST-1. Spleen cells (1 × 10^6^/ml) were labeled with CellTrace far-red proliferation dye (CTFR) and stimulated with 2.5 μg/ml TSST-1, 2.5 μg/ml concanavalin A (positive control), or left unstimulated (negative control). Bars show means ± SD of results for 3 mice. ****, *P* < 0.0001 between TSST-1 and unstimulated splenocytes by two-way ANOVA. (C) Serum cytokines 2 h and 6 h after i.p. injection of 80 μg TSST-1 in HLA-DQ8 or C57BL/6 mice. Values to the right of the dashed line refer to the *y* axis on the right. Medians and 5th, 25th, 50th, 75th, and 95th centiles for five individual mice are shown. *, *P* < 0.05 by Mann-Whitney U test between HLA-DQ8 and C57BL/6 mice treated with TSST-1. IFNγ, gamma interferon; IL-6, interleukin 6; IL12p70, interleukin 12 (p70); IL-17, interleukin 17; KC, CXCL1; MCP-1, monocyte chemotactic protein 1.

The proliferation of mouse spleen cells in response to superantigens was assessed to determine the superantigen sensitivity of transgenic mice in comparison to that in wild-type mice to recapitulate human immune responses to superantigens, such as those which occur in TSS. Spleen cells of HLA-DQ8 transgenic mice were markedly more sensitive to purified TSST-1 than spleen cells from wild-type C57BL/6 mice (Fig. 1A). Indeed, HLA-DQ8 spleen cells were more sensitive to all staphylococcal superantigens tested than cells from either HLA-DR4 or wild-type mice (see Fig. S2 in the supplemental material). Because of this, all further experiments were performed using HLA-DQ8 mice.

The response of HLA-DQ8 splenocytes to superantigens was compared to that of human peripheral blood mononuclear cells (PBMCs). HLA-DQ8 mice were sensitive to TSST-1 at micromolar concentrations and to SEB at nanomolar concentrations. Human PBMCs were sensitive at picomolar concentrations to both superantigens (Fig. S3).

Following coculture, TSST-1 expanded HLA-DQ8 mouse spleen cell T-cell receptor (TCR) Vβ subsets TCR Vβ3 and TCR Vβ13 (Fig. 1B). HLA-DQ8 mice treated with TSST-1 intraperitoneally (i.p.) had elevated levels of the serum cytokines interleukin 6 (IL-6), KC (CXCL1), IL-12p70, IL-17, and MCP-1 at 2 h and gamma interferon (IFN-γ) at 6 h compared to levels in wild-type C57BL/6 control mice (Fig. 1C). Levels of other cytokines tested did not differ between groups (Table S2).

### Modeling soft tissue infection in HLA-DQ8 mice.

**FIG 2:**
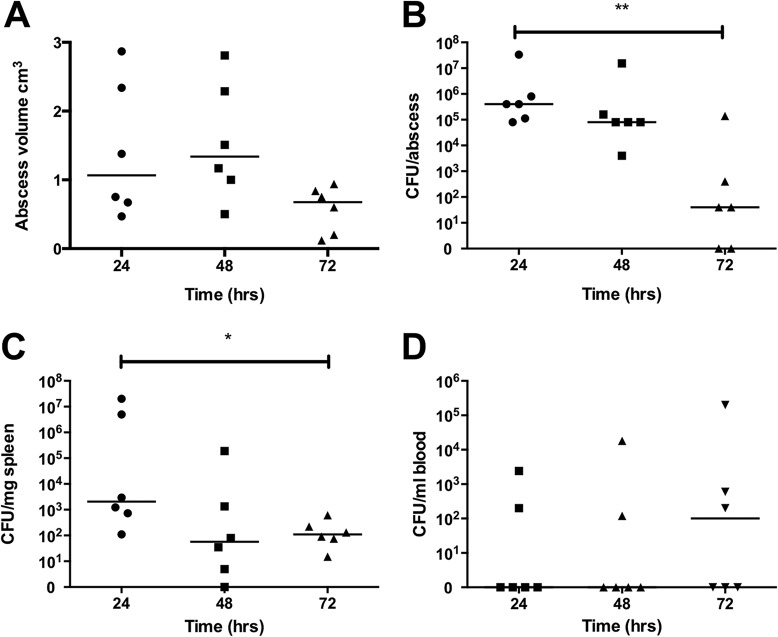
Bacteriology of TSST-1-producing S. aureus infection in HLA-DQ8 mice. Mice were infected subcutaneously with *tst*-positive CC30 MSSA strain HSS357 (1 × 10^9^ CFU). At 24, 48, and 72 h, mice were culled and abscess dimensions were measured (A), pus was extracted and plated for CFU quantification (B), spleens were extracted, homogenized, and plated for CFU counting (C), and blood cultures were taken by cardiac puncture (D). Median values are shown from six HLA-DQ8 mice per group. *, *P* < 0.05; **, *P* < 0.01 by Mann-Whitney U test of HLA-DQ8 groups compared between 24 h and 72 h.

Having demonstrated responsiveness to TSST-1, HLA-DQ8 transgenic mice were infected subcutaneously (s.c.) with TSST-1-producing S. aureus, and groups were euthanized at 24, 48, and 72 h. By 24 h, there was visible abscess formation at the inoculation site. Abscess volume and bacterial load decreased by 72 h. Bacteria disseminated to the spleen at all time points in 17/18 mice, though spleen bacterial loads decreased during infection. Two to three mice in each group had detectable bacteremia at each time point (Fig. 2). Weight loss was maximal 24 h following infection (median, 9.3%; range, 0 to 15.9%).

**FIG 3:**
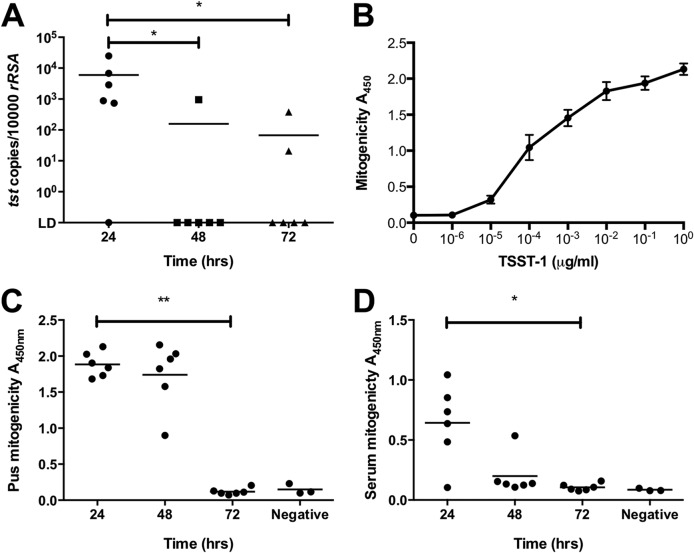
Local *tst* transcription and mitogenicity of pus and serum in TSST-1-producing S. aureus infection in HLA-DQ8 mice. Groups of 6 mice were infected subcutaneously with *tst*-positive S. aureus strain HSS357 (1 × 10^9^ CFU). At 24, 48, and 72 h, groups were culled and abscess pus was extracted. (A) Copies of *tst* transcripts were measured by quantitative real-time PCR per 10,000 copies of *rrsA.* (B) Proliferation of human PBMCs incubated with 0 to 1 μg/ml TSST-1 in triplicate, measured by BrdU uptake. Human PBMCs were incubated with pus (C) and serum (D) obtained at 24-h time points after infection. Proliferation was measured by BrdU uptake. Data and medians are shown for 6 individual HLA-DQ8 mice per group. Negative, tissue culture medium (RPMI 1640) alone. *, *P* < 0.05; **, *P* < 0.01 by Mann-Whitney U test of HLA-DQ8 groups compared to one another on each day of infection.

*tst* mRNA transcripts in pus obtained from the abscess were detectable in 5/6 mice at 24 h, 1/6 mice at 48 h, and 2/6 mice at 72 h and were maximal at 24 h (Fig. 3A). Due to the use of abscess samples for RNA and other analyses, measurement of TSST-1 protein was undertaken for just one mouse at each time point; TSST-1 protein was, however, detected by Western blotting in the 24-h pus sample (8 μg/ml) but not at 48 or 72 h. Human PBMCs were sensitive to TSST-1 at nanomolar concentrations (Fig. 3B). Pus recovered from abscesses demonstrated sustained mitogenic activity toward human PBMCs at 24 and 48 h, but not at 72 h, when diluted 1:100. Strong mitogenic activity toward human PBMCs was also detected in all sera at 24 h despite infrequent bacteremia, consistent with the presence of superantigen in the mouse serum (Fig. 3C and D).

Serum cytokines and chemokines were maximal 24 h postinfection, consistent with the findings of purified TSST-1 challenge. IL-6, IFN-γ, KC, MCP-1, MIP-1α, and granulocyte colony-stimulating factor (G-CSF) were raised in infected transgenic mice, unlike with control HLA-DQ8 mice inoculated with phosphate-buffered saline (PBS) alone (Table S3).

On histological analysis of abscess sections from single mice, bacteria were detected on each day of infection, accompanied by heavy subcutaneous infiltration by neutrophils, with inflammation (Fig. S4).

### Draining lymph node involvement during S. aureus infection.

**FIG 4:**
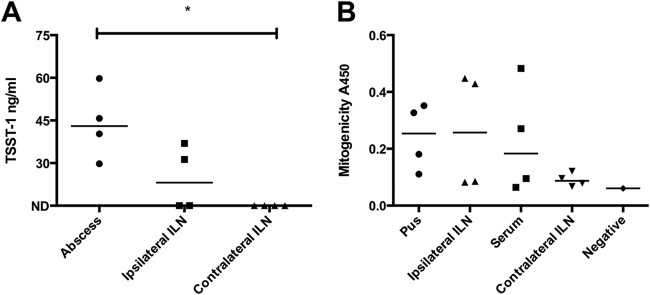
Tissue TSST-1 production and mitogenicity during infection with TSST-1-producing S. aureus in HLA-DQ8 mice. HLA-DQ8 mice were infected subcutaneously with *tst*-positive CC30 MSSA strain HSS357 (1 × 10^9^ CFU). (A) Mice were culled at 24 h after infection, and TSST-1 protein present in the abscess and inguinal lymph nodes (ILN) was measured by Western blotting. TSST-1 protein of known concentration was measured to quantify the amount of TSST-1 by densitometry. (B) Human PBMC responses to pus, inguinal lymph nodes, and sera from infected mice. Proliferation was measured by BrdU uptake. Negative, tissue culture medium (RPMI 1640) alone. ND, not detected. *, *P* < 0.05.

To determine whether S. aureus infection involved draining inguinal lymph nodes, four HLA-DQ8 mice infected with the CC30 *tst*-positive S. aureus strain HSS357 were euthanized 24 h following infection and dissected, with careful removal of inguinal lymph nodes. Bacteria were detected in the subcutaneous abscess (median, 1.3 × 10^7^ CFU; range, 0 to 2.8 × 10^7^ CFU/abscess), the ipsilateral inguinal lymph node (median, 8.5 CFU; range, 0 to 1.4 × 10^4^ CFU/lymph node), and spleen (median, 4.0 × 10^−1^ CFU; range, 2.0 × 10^−1^ to 2.6 × 10^3^ CFU/mg spleen), but not the contralateral inguinal lymph node or blood. The greatest bacterial burden was in the subcutaneous abscess, but there was also abscessation in the ipsilateral inguinal lymph nodes of all mice. TSST-1 was detected at the highest level in pus from the subcutaneous abscesses of all four mice and was detected in the ipsilateral inguinal lymph nodes from 2/4 mice but not in any contralateral inguinal lymph node (Fig. 4A). Mitogenicity was elicited (in descending order of magnitude) from the subcutaneous abscess pus, ipsilateral inguinal lymph node, and serum from infected mice (Fig. 4B), consistent with the presence of superantigen.

### Antibiotic impact on lesion size and superantigen toxin production.

**FIG 5:**
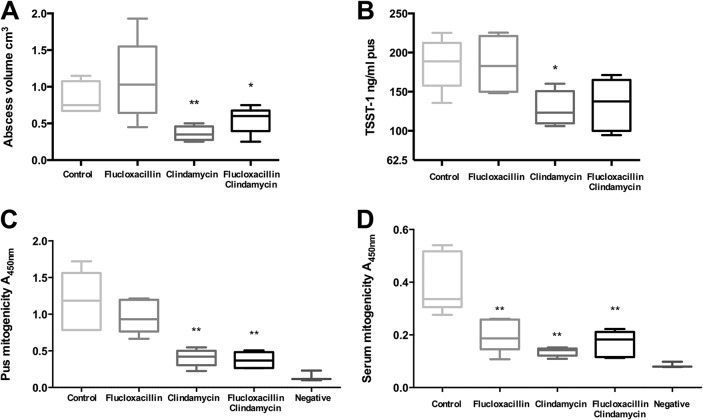
Antibiotics impact abscess volume, TSST-1 production, and mitogenicity during infection with TSST-1-producing S. aureus in HLA-DQ8 mice. HLA-DQ8 mice were infected subcutaneously with *tst*-positive CC30 MSSA strain HSS357 (1 × 10^9^ CFU). At 24 h, mice received flucloxacillin, clindamycin, flucloxacillin and clindamycin, or 100 μl PBS i.p. (as a control) and were culled at 30 h. (A) Abscess dimensions were measured. (B) TSST-1 protein present in abscesses was measured by Western blotting. TSST-1 protein of known concentration was also measured to quantify the amount of TSST-1 by densitometry. (C and D) Human PBMC responses to pus (C) and serum (D) from infected mice were measured. Proliferation was measured by BrdU uptake. Negative, tissue culture medium (RPMI 1640) alone. Medians and 5th, 25th, 50th, 75th, and 95th centiles are shown for five individual mice. *, *P* < 0.05; **, *P* < 0.01 by Mann-Whitney U test comparing different antibiotic regimens with the control.

S. aureus-infected HLA-DQ8 mice were treated with a single dose of flucloxacillin (FCX), clindamycin (CLD), FCX-CLD, or sterile PBS at 24 h postinfection and were euthanized at 30 h, i.e., 6 h after antibiotic or PBS administration, to assess the impact of antibiotics on TSST-1 production and the host immune response. Within 6 h of antibiotic administration, mice treated with CLD-containing regimens had smaller abscesses and reduced local TSST-1 production compared to those of mice treated with PBS or FCX alone (Fig. 5A and B). Accordingly, there was a clear reduction in pus and serum sample mitogenicity (Fig. 5C and D). The pus sample was not subjected to quantitative real-time PCR (qRT-PCR) of *tst* transcripts due to previously low levels of transcript detection.

**FIG 6:**
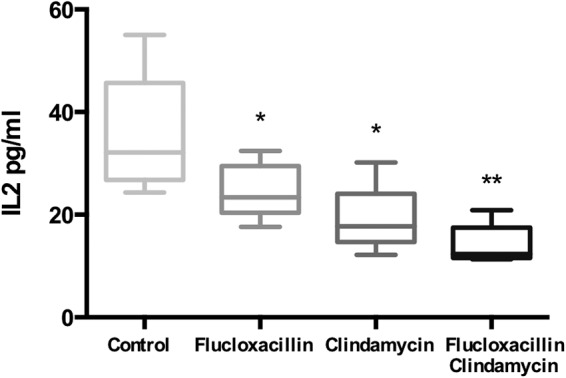
Interleukin-2 levels after antibiotic administration to HLA-DQ8 mice infected with TSST-1-producing S. aureus. HLA-DQ8 mice were infected subcutaneously with *tst*-positive S. aureus strain HSS357 (1 × 10^9^ CFU). At 24 h, mice received flucloxacillin, clindamycin, flucloxacillin and clindamycin, or 100 μl PBS i.p. (as a control) and were culled at 30 h. Serum was collected by cardiac puncture and analyzed by immunoassay. Medians and 5th, 25th, 50th, 75th, and 95th centiles are shown for five individual mice. *, *P* < 0.05; **, *P* < 0.01 by the Mann-Whitney U test compared to control mice injected with PBS alone.

**FIG 7:**
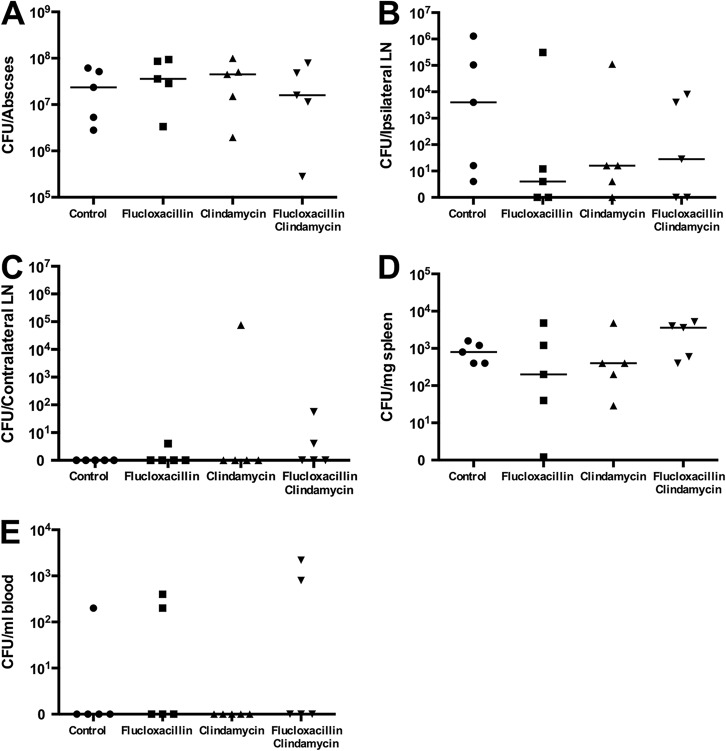
Bacteriology of the antibiotic impact on HLA-DQ8 mice infected with TSST-1-producing S. aureus. HLA-DQ8 mice were infected subcutaneously with *tst*-positive S. aureus strain HSS357 (1 × 10^9^ CFU). At 24 h, mice received flucloxacillin, clindamycin, flucloxacillin and clindamycin, or 100 μl PBS i.p. (as a control) and were culled at 30 h. (A) Abscess pus was extracted and plated for CFU counting; (B and C) ipsilateral/contralateral inguinal lymph nodes (LN) were excised, homogenized, and plated for CFU counting; (D) spleens were extracted, homogenized, and plated for CFU counting; (E) blood cultures were taken by cardiac puncture. Data and medians are shown for 5 individual mice per group.

Cytokine differences between control and antibiotic-treated groups were negligible; however, the level of IL-2 was reduced in mice treated with CLD-containing regimens compared to those in mice treated with PBS or FCX, consistent with reduced T-cell stimulation (Fig. 6). The single antibiotic dose did not impact the numbers of CFU detected in the abscess, lymphoid organs, or blood compared with those detected in mice given PBS (Fig. 7). In particular, CLD did not confer any additional benefit with regard to bacteriological clearance during this short treatment time.

## DISCUSSION

We describe an S. aureus subcutaneous-abscess model in HLA-DQ8 transgenic mice. These mice demonstrated sensitivity to TSST-1, which expanded the murine T-cell receptor Vβ subsets TCR Vβ3 and TCR Vβ13. S. aureus disseminated to the draining inguinal lymph nodes and spleen, while TSST-1 production was detected in not only the subcutaneous abscess but also the draining inguinal lymph node, signaled by mitogenic activity in the abscess pus, the ipsilateral inguinal lymph node, and serum. Clindamycin-containing antibiotic regimens reduced abscess volume, TSST-1 production, and the overall mitogenic activity of the lesion within just 6 h of a single treatment.

Experimental comparison of infections with different strains of transgenic mice was not possible in this study. It was notable that HLA-DQ8 mice appeared to be more sensitive to TSST-1 than HLA-DR4 mice *in vitro*, despite previous work indicating that TSST-1 binds to HLA-DR molecules with levels of affinity greater than (9) or equal to (10) its levels of affinity to HLA-DQ molecules. Polymorphisms in HLA-DR are also known to affect transgenic-mouse responses to TSST-1 (11). It is likely that differential expression of the HLA transgenes and endogenous H2 or responding T-cell subset repertoires may explain the enhanced responses in HLA-DQ8 mice, as we noted that responsiveness to concanavalin A (ConA) and medium alone was also greater in HLA-DQ8 mice. Notwithstanding the *in vitro* findings, infection with a CC30 S. aureus isolate that produces a high level of TSST-1 led to marked suppuration, abscess formation, and cytokine production.

We believe that the suppuration observed was in part related to TSST-1 responsiveness. It was not possible to evaluate an isogenic, *tst*-deficient strain to determine which of the observed effects were due solely to TSST-1; the CC30 lineage of S. aureus is challenging to transform, although recent tools may now allow for its manipulation (12). HSS357 carried (aside from *tst*) *sei* and *seg*, which are found in the enterotoxin gene cluster (*egc*). The *egc* gene cluster is widespread in S. aureus strains (13) and unlikely to have any specific association with TSS.

The measurement of TSST-1 production *in vivo* provides valuable contextual information to inform future superantigen research. In some experiments, we detected higher levels of TSST-1 in subcutaneous abscess pus than could be detected during broth culture of the same strain (4) and higher levels than previously reported in the abscesses and kidney extracts of mice infected with TSST-1-producing S. aureus (14, 15), consistent with upregulation of superantigen production during suppurative infection. Microbial spread and suppuration were noted in the ipsilateral inguinal lymph node, a tissue rich in superantigen-sensitive T cells, and TSST-1 protein was detected here too 24 h after infection. The detection of bacteria and TSST-1 in ipsilateral, but not contralateral, inguinal nodes is consistent with transit in afferent lymphatic vessels rather than blood. During infection, abscessation was present in all the ipsilateral inguinal lymph nodes, including those without detectable viable bacteria, as S. aureus may have been killed by the neutrophils creating the pus. We cannot determine whether TSST-1 was produced within the lymph node by S. aureus or transferred from the site of infection via lymphatics. The proximity of TSST-1-producing S. aureus to such lymphoid tissues may be pivotal to events occurring during TSS; the extent to which this occurs in clinical disease is unknown. It is widely believed that TSS results from the systemic dissemination of superantigens into the bloodstream and consequent interaction with leukocytes in the lympho-reticular system; in mice, the spleen is known to act as a major source of cytokines following systemic superantigen administration (16). Our results raise the possibility that superantigen exposure within secondary lymphoid organs, such as the lymph nodes, may contribute to the cytokine storm underlying TSS pathogenesis during infection. Previous studies using HLA transgenic mice have reported IL-6, IFN-γ, and IL-2 serum responses in HLA-DQ8 mice exposed to aerosolized SEB (17) and IL-6 and IFN-γ production by HLA-DR3 spleen cells exposed to SEB (18). We detected cytokine responses to a bolus of toxin and to infection; IL-6, IFN-γ, KC, and MCP-1 were the most consistent markers of inflammation.

Lethal shock was not observed or expected in this model; we note that sensitization agents were not used and that bacterial clearance occurred over the 72-h experimental period. Thus, this model does not replicate TSS as observed in humans but served to elucidate potential pathways of TSST-1 production, dissemination, and abscess progression in a model that reproduces some of the immunological responses to staphylococcal infection seen in humans. Previous models of TSS in HLA-DR1 mice required d-galactosamine pretreatment to elicit liver failure, an event that is entirely related to tumor necrosis factor (TNF)-induced hepatocyte apoptosis in the d-galactosamine setting, while SEB alone results in cytokine release only (16). Changes in serum cytokine levels were observed in both HLA-DQ6 and HLA-DQ8 mice at 4 h following SEB and streptococcal pyrogenic exotoxin A (SPEA) challenge (19). In our study, weight loss, *tst* transcripts, TSST-1 protein, and cytokines were maximal at 24 h following infection, consistent with a marked systemic inflammatory response that might be like that observed in nmTSS.

TSST-1 expanded TCR Vβ subsets 3 and 13 in HLA-DQ8 mouse splenocytes. TSST-1-induced TCR Vβ15 and -17 subset expansion, as was previously reported in earlier murine studies, could not be evaluated in the current study, as the assay used did not detect them (20, 21). Further work to determine whether TSST-1 results in specific T-cell Vβ subset expansion and cytokine release within lymphoid organs in the context of S. aureus infection would provide novel insight into nmTSS pathogenesis.

Notwithstanding findings in local lymph nodes, the mitogenicity assays strongly pointed to the presence of superantigen in the sera of infected mice. While we could not directly quantify TSST-1 in serum, parallel standard TSST-1 bioactivity assays yielded data suggesting that 1 to 10 pg/ml of TSST-1 was present in the blood. TSST-1 at 0.2 pg/ml is reported to cause half-maximum proliferation of human T cells (2). We cannot rule out the possibility that low levels of S. aureus were present in the blood (limit of detection, 200 CFU/ml). However, the absence of a detectable bacteremia supports the assertion that TSST-1 may disseminate from the initial infection site to systemic circulation either by transcytosis across cellular barriers to reach the blood (22) or via the lymphatic system, enabling activation of T cells distant to the site of infection.

Current TSS treatment recommendations advise a combination of β-lactam and lincosamide antibiotics, until culture results are known (5). This is based on *in vitro* studies, extrapolation from observational studies of streptococcal TSS, and *in vivo* evaluations of the effects of protein synthesis inhibitors in rabbit models of pneumonia using Panton-Valentine leukocidin-producing S. aureus (23). There is a lack of published *in vivo* data on the effect of clindamycin on TSST-1 production in any infection model. We elected to evaluate this effect and whether using clindamycin at the outset of TSS management might impact disease progression. We chose to treat a time point when *tst* transcripts and TSST-1 protein were maximal and cytokines detectable. Unsurprisingly, a single dose of antibiotic did not reduce abscess bacterial burden, consistent with previous findings (24). Notably, however, mice treated with clindamycin-containing regimens had smaller abscesses, reduced TSST-1 production, and diminished mitogenicity of pus and serum compared to those of mice treated with other regimens. This is the first work to demonstrate the superantigen-inhibitory effects of clindamycin *in vivo*. The findings suggest that clindamycin may have an indirect effect on disease and abscess progression, potentially by reducing TSST-1 synthesis, despite little measurable effect on bacterial counts by this model. Previous reports do suggest that abscessation may be enhanced by superantigens; hepatic abscesses are known to develop in HLA transgenic mice exposed to SEA-producing S. aureus (25). Although clindamycin can exert an inhibitory effect on superantigen-induced host cytokine production *in vitro* (26), serum cytokine levels in our study did not demonstrate a clear antibiotic effect, perhaps due to the timing of analysis following one antibiotic dose.

Our findings support the adjunctive use of clindamycin to modify disease progression in the treatment of suspected staphylococcal TSS, to reduce superantigen toxin production more rapidly, and to potentially reduce abscessation. Further studies are required to increase our understanding of TSS pathogenesis and the role of lymph node superantigen expression and to explore the efficacy of treatment with other immune modulators, such as intravenous immunoglobulin, to limit the lethal potential of this syndrome.

## MATERIALS AND METHODS

### Animals.

Female HLA class II transgenic mice on a C57BL/6 background carrying genomic constructs for HLA-DQA1*0301/HLA-DQB*0302 (DQ8), HLA-DRA1*0101/HLA-DRB1*0401 (DR4, H2 Aβ0; Taconic Farms) (8, 27, 28), and C57BL/6 mice (Charles River, UK) that were 8 to 14 weeks old were used in accordance with a UK Home Office-approved project license following assessment by the Imperial College Ethical Review Process. Mice were acclimatized for 1 week prior to use.

### Bacterial culture.

HSS357, a clinical *tst*-positive CC30 MSSA strain that caused TSS, was selected based on highest *in vitro* TSST-1 production (187 ng/ml following overnight culture in 5 ml of brain heart infusion [BHI] broth) among clinical *tst*-positive CC30 MSSA isolates causing TSS (4). Overnight culture of strain HSS357 in 50 ml BHI yielded 400 ng/ml of TSST-1, although transcription of *tst* peaked at 8 h and diminished thereafter (see Fig. S1 in the supplemental material). HSS357 was sensitive to all antibiotics, including clindamycin and flucloxacillin, with the exception of penicillin. Antibiotic MICs were determined by British Society for Antimicrobial Chemotherapy methods (http://www.bsac.org.uk) and interpreted in accordance with European Committee on Antimicrobial Susceptibility Testing guidelines (http://www.eucast.org). HSS357 carried the superantigen genes *seg* and *sei* in addition to *tst*, determined by toxin gene profiling (*sea* to *see*, *seg* to *sej*, *tst*, and *pvl*) by multiplex PCR (29, 30).

10.1128/mSphere.00665-19.1 FIG S1 Time course of TSST-1 production and *tst* expression by *tst*-positive CC30 MSSA strain HSS357. *tst*-positive CC30 MSSA strain HSS357 was cultured (*n* = 1) for 24 h in 50 ml BHI. Supernatants were collected and concentrated (25-fold), and bacterial pellets were collected at regular intervals. (A) TSST-1 production by HSS357. Proteins were separated by SDS-PAGE and transferred to a polyvinyl difluoride (PVDF) membrane. TSST-1 protein was detected with rabbit ant-TSST-1 (1:10,000) and HRP-conjugated anti-rabbit (1:50,000). TSST-1 protein of known concentration was also measured to quantify the amount of TSST-1 by densitometry. (B) Copies of *tst* transcripts were measured by quantitative real-time PCR and normalized to the number of *tst* copies per 10,000 copies of *rrsA.* Download FIG S1, TIF file, 0.1 MB. Copyright © 2019 Sharma et al. 2019 Sharma et al. This content is distributed under the terms of the Creative Commons Attribution 4.0 International license.

10.1128/mSphere.00665-19.2 FIG S2 Sensitivity of HLA-DQ8, HLA-DR4, and C57BL/6 spleen cells to staphylococcal superantigens. Spleen cells (1 × 10^6^/ml) from HLA-DQ8, HLA-DR4/Aβ0, and C57BL/6 mice were exposed to 0 to 10 μg/ml of TSST-1 (A), SEA (B), SEB (C), or SEC (D). Proliferation was measured by [^3^H]thymidine uptake. Levels of proliferation in the presence of 5 μg/ml ConA were as follows: 229,806 ± 48,570 CPM for C57BL/6, 373,349 ± 56,008 CPM for HLA-DQ8, and 207,280 ± 31,798 CPM for HLA-DR4 mice. Proliferation in the presence of medium only was as follows: 1,330 ± 264 CPM for C57BL/6, 15,392 ± 6,941 for HLA-DQ8, and 6,754 ± 9,969 CPM for HLA-DR4. There was a significant difference among HLA-DQ8 and C57BL/6 and HLA-DQ8 and HLA-DR4 medium-only values. Data are means ± SD of results from three individual mice. *, *P* < 0.05; **P < 0.01 between HLA-DQ8 and either HLA-DR4 or C57BL/6 by ANOVA. Filled squares represent HLA-DQ8 mice, filled triangles represent HLA-DR4 mice, and filled circles represent C57BL/6 mice. Download FIG S2, TIF file, 0.3 MB. Copyright © 2019 Sharma et al. 2019 Sharma et al. This content is distributed under the terms of the Creative Commons Attribution 4.0 International license.

10.1128/mSphere.00665-19.3 FIG S3 Comparison of HLA-DQ8 splenocyte and human peripheral blood mononuclear cell proliferative responses to superantigens. Human PBMCs and spleen cells from HLA-DQ8 mice were exposed to 0 to 10 μg/ml TSST-1 (A) or SEB (B). Proliferation was measured by [^3^H]thymidine uptake. Data are means ± SD of results from three individual mice and one healthy human donor. **, *P* < 0.01; ****, *P* < 0.0001 between HLA-DQ8 and donor by ANOVA. Download FIG S3, TIF file, 0.1 MB. Copyright © 2019 Sharma et al. 2019 Sharma et al. This content is distributed under the terms of the Creative Commons Attribution 4.0 International license.

10.1128/mSphere.00665-19.4 FIG S4 Histology of *tst*-positive CC30 MSSA infection in HLA-DQ8 transgenic mice. Mice were infected subcutaneously with *tst*-positive S. aureus strain HSS357 (1 × 10^9^ CFU) or injected subcutaneously with 100 μl of PBS as a control. Infected mice were euthanized at 24, 48, and 72 h. Tissue from the site of injection was dissected and then fixed in formalin, embedded into paraffin blocks, and stained with hematoxylin and eosin (H&E) or Gram stained. (A) Control, PBS-injected HLA-DQ8 mice. Magnification, ×40. (B) Infected HLA-DQ8 mouse H&E stain at 48 h. Magnification, ×40. The circled area represents heavy inflammatory infiltrate in subcutaneous tissue below the level of the muscle wall, with cocci. (C) Infected HLA-DQ8 mouse HE stain at 48 h. Magnification, ×400. Heavy inflammatory infiltrate in subcutaneous tissue below the level of the muscle wall, with cocci. (D) Infected HLA-DQ8 mouse Gram stain at 24 h. Magnification, ×1,000. Subcutaneous tissue with dense focal inflammation around Gram-positive cocci (circled). Sections are from one mouse from each day of infection. Download FIG S4, TIF file, 1.0 MB. Copyright © 2019 Sharma et al. 2019 Sharma et al. This content is distributed under the terms of the Creative Commons Attribution 4.0 International license.

For *in vivo* administration, HSS357 was cultured overnight in BHI broth at 37°C with agitation at 200 rpm and then centrifuged, washed, and resuspended in sterile phosphate-buffered saline (PBS). Inocula, pus, and tissue samples for culture were plated onto Luria broth (LB) agar and incubated overnight at 37°C prior to quantification of CFU per milliliter.

### Superantigen sensitivity in transgenic mice.

Spleen cells (1 × 10^6^/ml) from HLA-DQ8, HLA-DR4, and C57BL/6 mice were prepared in RPMI 1640 medium (Invitrogen, Hemel Hempstead, UK) supplemented with 10% fetal calf serum, 2 mM glutamine, 50 U/ml penicillin and streptomycin, and 0.01 mM mercaptoethanol and coincubated with 10 pg/ml to 10 μg/ml of highly purified TSST-1, SEA, SEB, or SEC (Toxin Technology, Sarasota, FL, USA) at 37°C for 48 h. Proliferation was measured after incorporation of 1.0 μCi/well of [^3^H]thymidine and an additional 16 h of incubation.

To assess TSST-1-induced T-cell receptor (TCR) Vβ subset expansion, HLA-DQ8 spleen cells (1 × 10^6^/ml) were labeled with CellTrace far-red proliferation dye (CTFR; Thermo Fisher Scientific, Hemel Hempstead, UK) and stimulated with 2.5 μg/ml TSST-1 at 37°C for 72 h. Murine spleen cells were (i) blocked with anti-mouse CD16/CD32, (ii) labeled with anti-CD3ε–phycoerythrin (PE) and anti-Vβ–fluorescein isothiocyanate (FITC) (against either Vβ subset 2, 3, 4, 5.1 and 5.2, 6, 7, 8.1 and 8.2, 8.3, 9, 10, 11, 12, 13, or 14) (mouse Vβ screening panel; BD Pharmingen), (iii) stained with 7-aminoactinomycin D (7-AAD) viability dye, and (iv) acquired on a FACSCalibur flow cytometer. Live CD3ε^+^ Vβ^+^ cells populations were gated, and proliferation was determined by the intensity of CTFR staining using Flow Jo v10.1 (Tree Star) and FCS Express v5 (De Novo Software).

To determine superantigen sensitivity *in vivo*, 80 μg of TSST-1 in 100 μl of PBS was administered intraperitoneally to HLA-DQ8 and C57BL/6 mice. Blood was taken by tail bleed at 2 h and by cardiac puncture at 6 h. Serum was separated and stored at –20°C for cytokine analysis.

### Bacterial infection.

HLA-DQ8 mice were infected subcutaneously on a shaved area of the right flank with 1 × 10^9^ CFU of *tst*-positive S. aureus in 100 μl sterile PBS or with PBS alone as a control. To investigate antibiotic impact, mice were given intraperitoneal flucloxacillin (FCX; 12.5 mg/kg of body weight), clindamycin (CLD; 10 mg/kg), or flucloxacillin with clindamycin (FCX-CLD, 12.5 mg/kg and 10 mg/kg, respectively) in 100 μl of PBS or 100 μl PBS as a control 24 h postinfection.

Mice were euthanized at various time points postinfection, blood was taken by cardiac puncture for CFU quantification, and sera were collected and stored at –20°C for cytokine analysis and mitogenicity assays. Abscess dimensions (height, width, and depth) were measured by a single observer using a mini-Vernier caliper for all experiments. Pus was excised by forceps at the time of dissection and stored in sterile Tris-EDTA buffer (10 mM Tris-HCl, pH 8, 1 mM EDTA). Aliquots of pus were taken for bacterial quantification, storage at –20°C for mitogenicity testing, RNA isolation (31), and local TSST-1 detection and quantification by immunoblotting. Spleens and left and right inguinal lymph nodes were homogenized in sterile PBS for CFU quantification, and lymph nodes were stored at –20°C for *ex vivo* mitogenicity testing.

### Expression of *tst in vitro* and *in vivo*.

One microgram of cDNA was synthesized from bacterial RNA treated with Turbo DNase (Ambion; Thermo Fisher) with Transcriptor reverse transcriptase (Roche, Basel, Switzerland) and random hexamer primers (Sigma, Dorset, UK). Quantitative real-time PCR (qRT-PCR) was performed using PCR primers for *tst* and the housekeeping gene *rrsA* (Table S1) with SYBR green JumpStart *Taq* ReadyMix (Sigma). Transcript copies were calculated by comparison with standard 10-fold concentrations of plasmid pCR2.1 (Invitrogen, Hemel Hempstead, UK) containing single copies of target genes (*tst* or *rrsA*) amplified alongside bacterial cDNA. Numbers of copies of sample *tst* transcripts were normalized to 10,000 copies of *rrsA.*

10.1128/mSphere.00665-19.5 TABLE S1 Primers used in this study. Download Table S1, DOCX file, 0.02 MB. Copyright © 2019 Sharma et al. 2019 Sharma et al. This content is distributed under the terms of the Creative Commons Attribution 4.0 International license.

10.1128/mSphere.00665-19.6 TABLE S2 Cytokine/chemokine production by C57BL/6 and HLA-DQ8 mice treated with intraperitoneal TSST-1. Download Table S2, DOCX file, 0.03 MB. Copyright © 2019 Sharma et al. 2019 Sharma et al. This content is distributed under the terms of the Creative Commons Attribution 4.0 International license.

10.1128/mSphere.00665-19.7 TABLE S3 Cytokine/chemokine production by HLA-DQ8 mice infected with TSST-1-producing S. aureus. Download Table S3, DOCX file, 0.03 MB. Copyright © 2019 Sharma et al. 2019 Sharma et al. This content is distributed under the terms of the Creative Commons Attribution 4.0 International license.

### Detection of TSST-1 by Western blotting.

Proteins were separated by 10% Bolt Bis-Tris Plus gel, transferred to nitrocellulose (Amersham Protran, GE Healthcare, Amersham UK), blocked, and then probed after incubation with rabbit anti-TSST-1 polyclonal antibody (Abcam, Cambridge, UK) and anti-rabbit horseradish peroxidase (HRP)-conjugated antibody (Life Technologies, Hemel Hempstead, UK) with an ECL Plus substrate detection system (Life Technologies). TSST-1 concentration in samples was determined by comparison with standard concentrations of TSST-1 simultaneously analyzed by densitometry (LabWorks, UVP, CA, USA). Samples below the detection limit were assigned half the value of the lowest standard concentration detected.

### Human T-cell proliferation assay.

Normal donor peripheral blood mononuclear cells (PBMCs) from anonymized consenting healthy donors were obtained from an approved subcollection of the Imperial College NHS Trust Tissue Bank (ICHTB reference R12023). PBMCs (1 × 10^6^/ml) were incubated in RPMI 1640 medium (Life Technologies) containing 10% fetal calf serum, 2 mM glutamine, and 50 U/ml of penicillin and streptomycin with a 1:100 dilution of murine pus or mouse serum at 37°C for 48 h. All measurements were performed as technical replicates in triplicate. T-cell proliferation was measured after incorporation of 1.0 μCi/well of [^3^H]thymidine and an additional 16 h of incubation or after T cells were labeled with 10 μm of BrdU (Roche, Welwyn Garden City, UK) and incubated for a further 4 h. The BrdU proliferation assay was used in place of [^3^H]thymidine incorporation during the study due to changes in the use of radioisotopes within the laboratory.

### Cytokine, chemokine, and growth factor measurement.

Serum cytokines were measured on a Bio-Rad Bio-Plex Luminex 200 system using a mouse 23-plex panel (Bio-Rad, CA, USA) that analyzed eotaxin, G-CSF, granulocyte macrophage CSF (GM-CSF), IFN-γ, IL-1α, IL-1β, IL-2, IL-3, IL-4, IL-5, IL-6, IL-9, IL-10, IL-12 (p40), IL-12 (p70), IL-13, IL-17A, KC, MCP-1, MIP-1α, MIP-1β, RANTES, and TNF-α. For analysis, samples below the detection limit were assigned half the value of the lowest level detected.

### Histopathology.

Tissue was dissected from randomly selected single HLA-DQ8 mice at each infection time point and fixed in formalin. Paraffin-embedded tissues were stained with hematoxylin and eosin or Gram’s stain and reviewed in a blind manner by a histopathologist (M. El-Bahrawy).

### Statistical analysis.

Data are stated as medians (ranges) or means ± standard deviations (SD). Data were analyzed with GraphPad Prism 6.0 (GraphPad Software, CA, USA) using analysis of variance (ANOVA), the Mann-Whitney U test, or an unpaired *t* test (two tailed) as indicated in the figure legends. Probability values of <0.05 were considered significant based on a two-tailed test.
