# Supplementary figures and images for: Toxic Shock Syndrome Toxin 1 Evaluation and Antibiotic Impact in a Transgenic Model of Staphylococcal Soft Tissue Infection
Source: mSphere. 2019 Oct 9;4(5):e00665-19. doi: 10.1128/mSphere.00665-19 (PMC6796978; doi:10.1128/mSphere.00665-19)

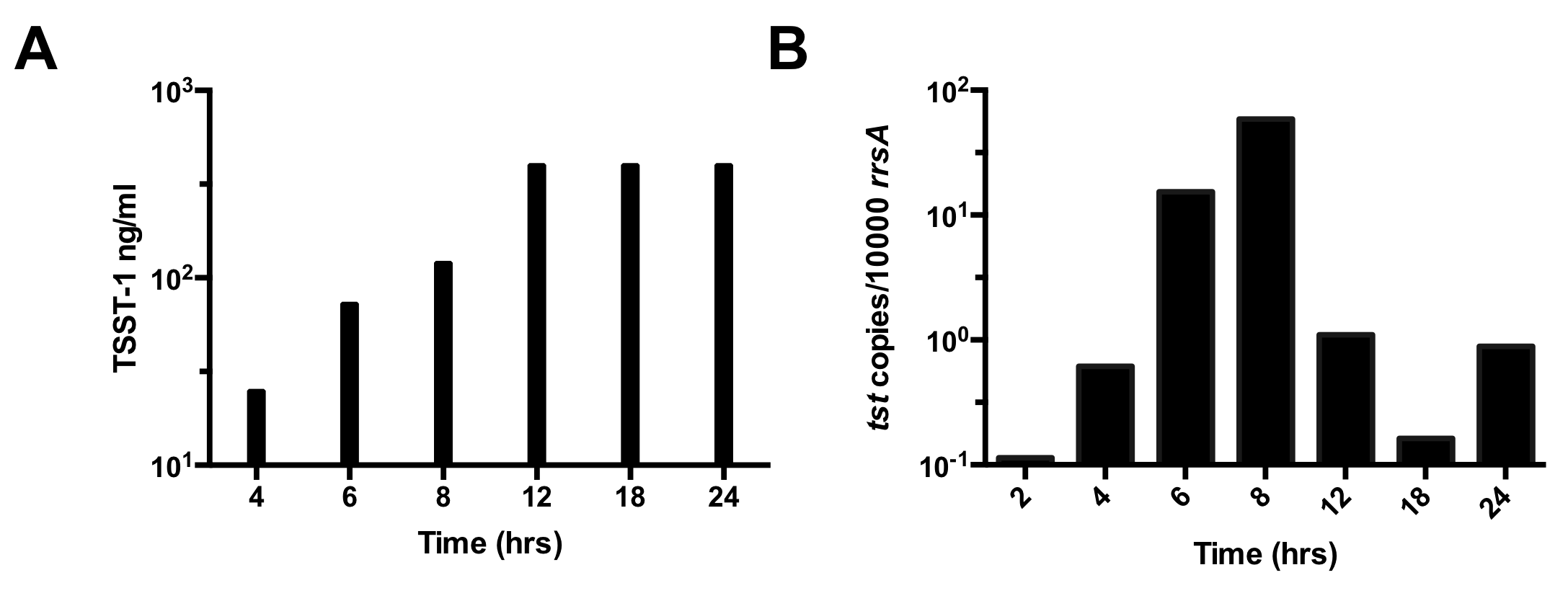

Supplement: FIG S1 [file mSphere.00665-19-sf001.tif]

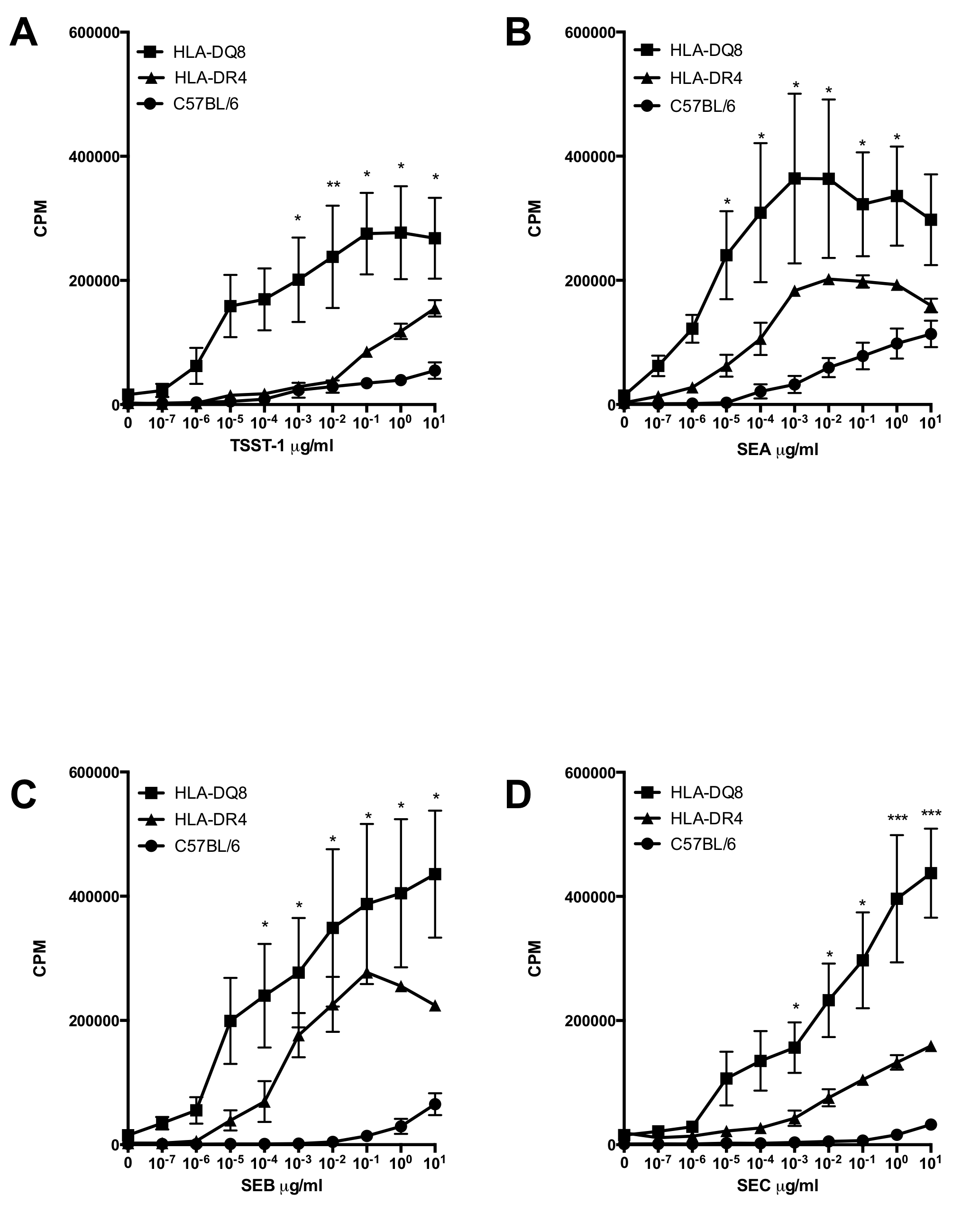

Supplement: FIG S2 [file mSphere.00665-19-sf002.tif]

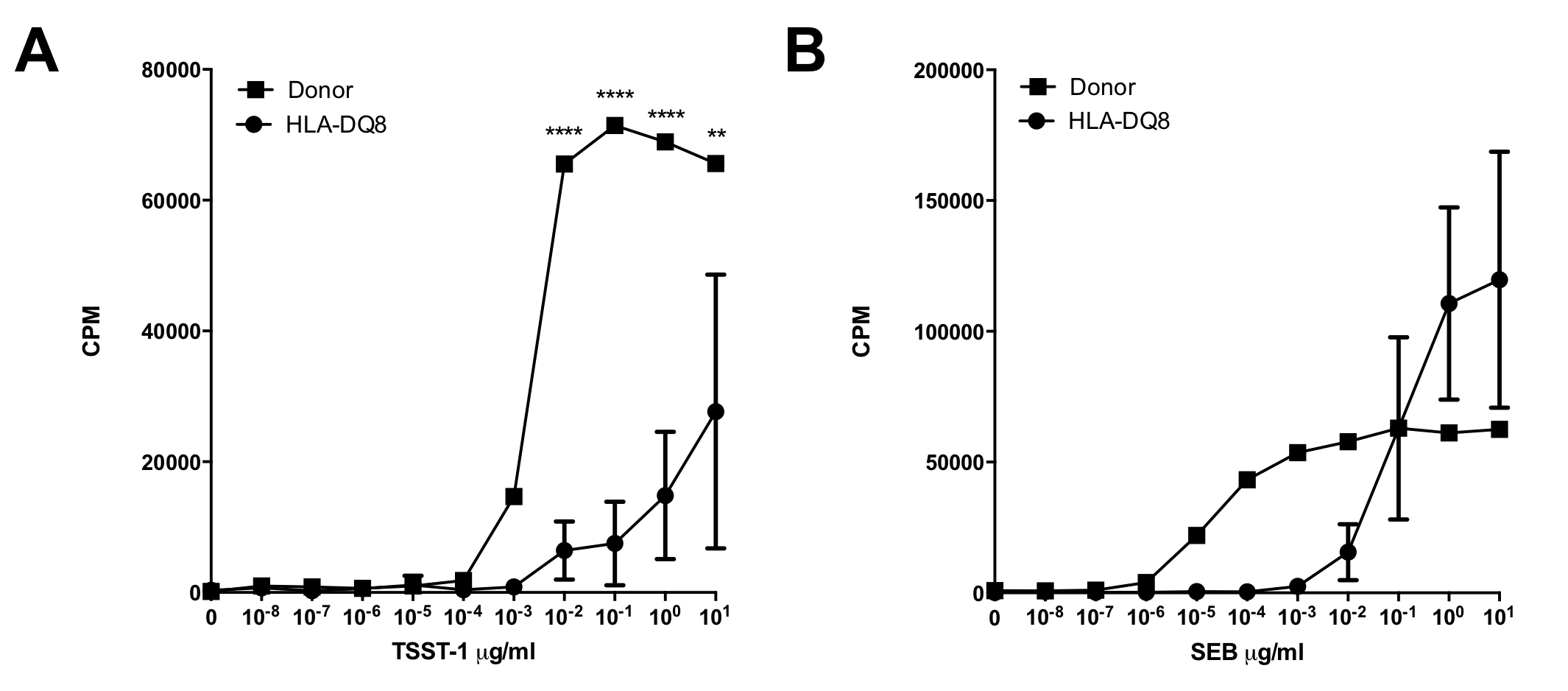

Supplement: FIG S3 [file mSphere.00665-19-sf003.tif]

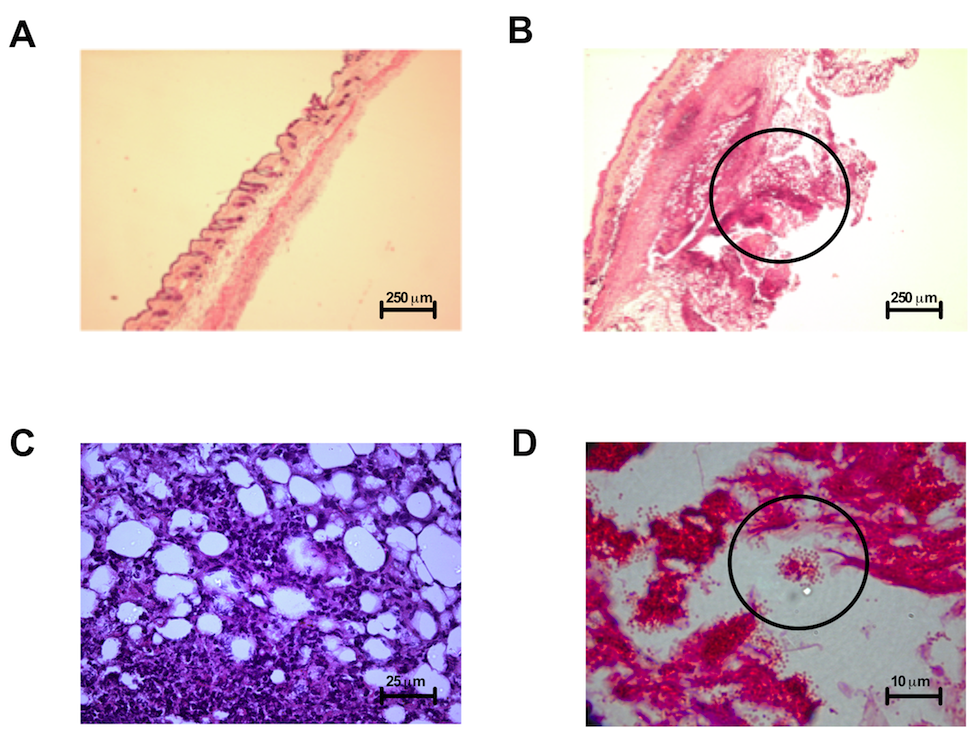

Supplement: FIG S4 [file mSphere.00665-19-sf004.tif]
