# Supplementary material for: Toxic Shock Syndrome Toxin 1 Evaluation and Antibiotic Impact in a Transgenic Model of Staphylococcal Soft Tissue Infection
Source: mSphere. 2019 Oct 9;4(5):e00665-19. doi: 10.1128/mSphere.00665-19 (PMC6796978; doi:10.1128/mSphere.00665-19)
Supplement: TABLE S1 [file mSphere.00665-19-st001.docx]

**Table S1. Primers used in this study**

| **Primer name** | **Primer sequence (5′-3′)** |
| --- | --- |
| *tst*- forward | CGAGTCCTTATTATAGCCCTGC |
| *tst*- reverse | GTTCCTTCGCTAGTATGTTGGC |
| *rrsA*-forward | AGCTTAGTTGCCATCATTAAGTTGG |
| *rrsA*-reverse | GTTGAGACTACAATCCGAACTG |
