# Supplementary material for: Toxic Shock Syndrome Toxin 1 Evaluation and Antibiotic Impact in a Transgenic Model of Staphylococcal Soft Tissue Infection
Source: mSphere. 2019 Oct 9;4(5):e00665-19. doi: 10.1128/mSphere.00665-19 (PMC6796978; doi:10.1128/mSphere.00665-19)
Supplement: TABLE S2 [file mSphere.00665-19-st002.docx]

**Table S2. Cytokine/chemokine production by C57BL/6 and HLA-DQ8 mice treated with intra-peritoneal TSST-1**

| **Cytokine***  **(pg/ml)** | **Time** | | | |
| --- | --- | --- | --- | --- |
|  | **2 h** | | **6 h** | |
|  | **C57BL/6**  **(n=5, median,(range))** | **HLA-DQ8**  **(n=5, median,(range))** | **C57BL/6**  **(n=5, median (range))** | **HLA-DQ8**  **(n=5, median,(range))** |
| IL1α | 6.21 (3.76-14.41) | 6.94 (0.88-16.93) | 5.81 (3.80-8.50) | 10.46 (4.45-10.70) |
| IL1β | 144.60 (127.70-164.30) | 144.60 (98.78-152.30) | 156.70 (142.30-167.60) | 147.90 (127.70-185.80) |
| IL2 | 898.80 (650.50-1157.00) | 1097.00 (904.60-2614.00) | 936.70 (499.20-1384.00) | 690.00 (248.80-1016.00) |
| IL3 | 2.50 (1.26-4.26) | 2.03 (1.56-2.97) | 5.42 (4.76-8.20) | 6.92 (2.50-9.48) |
| IL4 | 3.09 (3.09-3.09) | 3.09 (3.09-3.09) | 3.09 (3.09-3.33) | 3.09 (3.09-3.69) |
| IL5 | 31.99 (6.84-71.44) | 28.88 (6.44-58.48) | 267.90 (21.76-418.00) | 303.00 (138.10-462.80) |
| **IL6** | **45.24 (4.44-74.36)** | **193.30 (47.95-313.60)** | **15.57 (4.130-40.25)** | **17.33 (2.170-74.36)** |
| IL10 | 29.69 (20.80-41.62) | 21.76 (16.97-27.16) | 31.58 (29.05-40.37) | 31.58 (23.35-45.06) |
| IL12p40 | 396.60 (238.00-688.60) | 346.00 (130.30-452.30) | 584.00 (539.30-807.60) | 633.30 (172.70-1041.00) |
| **IL12p70** | **24.14 (13.24-44.26)** | **46.83(31.59-78.42)** | **36.62 (32.84-40.43)** | **31.59 (15.63-55.90)** |
| IL13 | 136.30 (113.20-153.80) | 132.20 (52.04-145.80) | 134.90 (54.88-204.60) | 137.70 (98.10-160.60) |
| **IL17** | **6.22 (6.22-11.25)** | **18.91 (8.04-45.61)** | **10.48 (6.97-11.25)** | **13.62 (7.35-17.61)** |
| Eotaxin | 104.00 (104.00-104.00) | 104.00 (104.00-104.00) | 104.00 (104.00-115.80) | 104.00 (104.00-104.00) |
| GCSF | 311.20 (171.70-898.80) | 342.30 (70.08-427.90) | 2458.00 (1541-3717.00) | 3116.00 (460.90-13559) |
| **IFNγ** | **3.96 (3.96-3.96)** | **3.96 (3.96-3.98)** | **5.64 (3.96-19.26)** | **19.67 (9.36-33.67)** |
| **KC** | **120. 70 (113.60-177.00)** | **271.70 (174.10-435.50)** | **108.60 (45.27-141.90)** | **48.40 (26.90-110.40)** |
| **MCP1** | **1855.00 (802.90-2512.00)** | **6032.00 (5359.99-7310.00)** | **883.60 (807.90-1473.00)** | **1238.00 (947.80-1739.00)** |
| MIP1α | 1.260 (0.84 – 7.81) | 3.52 (0.84-5.49) | 3.55 (1.36-4.90) | 4.27 (0.84-10.76) |
| MIP1β | 54.44 (34.75-81.41) | 61.30 (21.32-66.66) | 21.45 (13.78-22.90) | 20.79 (14.47-27.58) |
| RANTES | 21.30 (17.06-25.28) | 20.61 (7.47-27.30) | 69.71 (43.64-95.18) | 56.93 (11.06-227.30) |
| TNFα | 126.90 (129.60-182.00) | 121.40 (88.46-136.50) | 165.40 (115.90-193.00) | 136.50 (129.60-182.00) |

*Values for GMCSF and IL-9 were not obtained. Boldface indicates p<0.05 by Mann-Whitney U test between HLA-DQ8 and C57BL/6 mice treated with TSST-1.
