# Supplementary material for: Toxic Shock Syndrome Toxin 1 Evaluation and Antibiotic Impact in a Transgenic Model of Staphylococcal Soft Tissue Infection
Source: mSphere. 2019 Oct 9;4(5):e00665-19. doi: 10.1128/mSphere.00665-19 (PMC6796978; doi:10.1128/mSphere.00665-19)
Supplement: TABLE S3 [file mSphere.00665-19-st003.docx]

**Table S3. Cytokine/chemokine production by HLA-DQ8 mice infected with TSST-1 producing *S. aureus***

| **Cytokine***  **(pg/ml)** | **Time** | | | | | |
| --- | --- | --- | --- | --- | --- | --- |
|  | **24 h** | | **48 h** | | **72 h** | |
|  | **TSST-1 (n=6, median (range))** | **CTL (n=3, median (range))** | **TSST-1 (n=6, median (range))** | **CTL (n=3, median (range))** | **TSST-1 (n=6, median (range))** | **CTL (n=3, median (range))** |
| IL1α | 24.72 (50.63-55.37) | 8.12 (2.09-27.10) | 24.10 (6.96-36.59) | 6.58 (1.92-8.91) | 4.4 (1.30-5.90) | 2.96 (0.64-4.49) |
| IL1β | 119.79 (35.87-310.61) | 24.27 (24.27-43.35) | 61.36 (41.53-67.84) | 28.16 (24.27-92.13) | 166.03 (24.27-362.31) | 142.14 (73.53-266.40) |
| IL2 | 31.85 (14.82-47.73) | 2.83 (2.50-22.05) | 12.71 (1.80-52.02) | 6.96 (2.94-24.21) | 12.98 (2.07-73.31) | 20.49 (2.29-50.50) |
| IL3 | 45.68 (13.58-100.95) | 9.72 (0.64-39.40) | 38.00 (0.64-56.58) | 49.08 (1.68-76.01) | 36.73 (0.64-69.45) | 6.30 (1.68-43.83) |
| IL4 | 16.27 (4.56-29.97) | 4.56 (4.56-26.58) | 22.73 (4.56-24.61) | 11.53 (4.56-16.08) | 15.28 (4.56-31.74) | 4.65 (4.56-24.89) |
| IL5 | 28.13 (2.86-58.61) | 2.42 (1.38-2.61) | 9.23 (5.40-49.26) | 2.48 (0.98-4.41) | 11.45 (2.48-32.43) | 8.88 (2.23-10.42) |
| **IL6** | **166.44 (36.48-219.89)** | **2.81 (0.95-3.52)** | **63.51 (22.10-119.03)** | **11.24 (0.62-18.63)** | **13.36 (2.05-63.35)** | **4.31 (3.96-16.22)** |
| IL10 | 22.45 (13.30-64.61) | 13.30 (13.30-48.63) | 37.41 (13.30-74.07) | 16.82 (13.30-36.80) | 20.35 (13.30-69.85) | 13.30 (13.30-66.70) |
| IL12p40 | 227.06 (212.19-292.46) | 110.48 (96.44-196.50) | 334.52 (271.96-388.47) | 166.74 (17.45-183.42) | 297.68 (123.81-503.37) | 218.42 (152.88-417.36) |
| IL12p70 | 233.93 (78.21-513.35) | 33.19 (6.98-467.44) | 183.56 (6.98-412.26) | 193.73 (6.98-477.76) | 158.73 (6.98-522.97) | 51.95 (15.33-379.78) |
| IL13 | 93.01 (60.50-138.51) | 29.49 (29.49-33.28) | 56.68 (36.27-286.81) | 39.27 (29.49-103.86) | 93.18 (29.49-672.59) | 233.11 (33.28-247.71) |
| IL17 | 59.34 (21.83-94.83) | 20.89 (11.27-34.22) | 31.05 (3.54-57.08) | 59.08 (2.79-73.74) | 46.91 (1.67-84.68) | 15.90 (5.80-43.59) |
| Eotaxin | 542.75 (143.30-950.92) | 143.30 (143.30-161.47) | 899.89 (143.30-2675.14) | 143.30 (143.30-143.30) | 221.93 (143.30-981.39) | 450.23 (143.30-477.73) |
| **GCSF** | **10605.81 (2657.18-46025.63)** | **10.71 (6.02-42.11)** | **2211.09 (653.93-3837.14)** | **47.41 (2.40-73.87)** | **144.36 (41.98-669.06)** | **67.65 (12.21-91.09)** |
| GMCSF | 38.74 (23.17-134.45) | 36.01 (4.53-64.77) | 48.45 (23.17-114.96) | 58.25 (33.23-59.91) | 43.51 (23.17-106.97) | 23.17 (23.17-77.57) |
| **IFNγ** | **31.98 (12.82-51.79)** | **3.73 (3.73-3.73)** | **16.28 (1.54-36.22)** | **10.16 (3.73-10.88)** | **3.73 (3.73-35.97)** | **8.16 (3.73-16.85)** |
| **KC** | **232.18 (167.61-526.26)** | **17.18 (12.82-21.46)** | **78.02 (38.47-214.60)** | **21.89 (10.98-25.72)** | **36.95 (13.51-67.70)** | **21.25 (17.18-28.59)** |
| **MCP1** | **351.30 (225.75-553.50)** | **22.19 (14.95-24.55)** | **165.83 (65.55-266.89)** | **22.19 (9.29-34.34)** | **88.79 (32.64-268.75)** | **82.38 (23.37-103.98)** |
| **MIP1α** | **19.23 (14.22-35.45)** | **1.1 (0.83-15.30)** | **13.98 (4.23-20.29)** | **9.40 (0.83-10.93)** | **8.43 (0.83-18.24)** | **1.13 (0.83-13.80)** |
| MIP1β | 26.18 (12.80-59.48) | 5.37 (4.94-7.06) | 30.24 (15.07-73.63) | 14.92 (5.37-20.90) | 19.89 (5.72-92.50) | 25.17 (5.37-45.29) |
| RANTES | 17.99 (4.61-21.82) | 5.91 (4.62-10.81) | 10.77 (1.89-17.33) | 6.33 (1.43-11.54) | 11.78 (0.82-21.46) | 9.48 (4.08-13.67) |
| TNFα | 500.14 (312.37-1459.77) | 107.53 (97.63-270.77) | 892.01 (37.02-1146.30) | 442.48 (55.34-488.28) | 351.70 (24.68-1446.99) | 399.38 (109.52-833.69) |

*Values for IL-9 were not obtained. Boldface indicates p<0.05 by unpaired t-test (two-tailed) between HLA-DQ8 mice infected with *S. aureus* or control HLA-DQ8 mice inoculated with phosphate buffered saline.
